# AI, agentic models and lab automation for scientific discovery — the beginning of scAInce

**DOI:** 10.3389/frai.2025.1649155

**Published:** 2025-08-29

**Authors:** Thomas Hartung

**Affiliations:** ^1^Doerenkamp-Zbinden-Chair for Evidence-Based Toxicology, Department of Environmental Health and Engineering, Johns Hopkins Bloomberg School of Public Health and Whiting School of Engineering, Baltimore, MD, United States; ^2^Department of Biology, University of Konstanz, Konstanz, Germany

**Keywords:** generative artificial intelligence, scientific discovery, self-driving laboratories, scAInce paradigm, predictive toxicology, microphysiological systems, AI governance

## Abstract

Until recently, the conversation about generative artificial intelligence in science revolved around the textual prowess of large language models such as GPT-3.5 and the promise that they might one day draft a decent literature review. Since then, progress has been nothing short of breathtaking. We now find ourselves in the era of multimodal, *agentic* systems that listen, see, speak and act, orchestrating cloud software and physical laboratory hardware with a fluency that would have sounded speculative in early 2023. In this review, I merge the substance of our 2024 white paper for the World Economic Forum Top-10-Technologies Report with the latest advances through mid-2025, charting a course from automated literature synthesis and hypothesis generation to self-driving laboratories, organoid intelligence and climate-scale forecasting. The discussion is grounded in emerging governance regimes—notably the European Union Artificial Intelligence Act and ISO 42001—and is written from the dual vantage-point of a toxicologist who has spent a career championing robust, humane science and of a field chief editor charged with safeguarding scholarly standards in *Frontiers in Artificial Intelligence*. I argue that research is entering a “co-pilot to lab-pilot” transition in which AI no longer merely interprets knowledge but increasingly *acts upon it*. This shift promises dramatic efficiency gains yet simultaneously amplifies concerns about reproducibility, auditability, safety and equitable access.

## 1 Introduction

In 2023, the World Economic Forum (WEF) has embraced Artificial Intelligence-Facilitated Healthcare by creating a Transformative Map in collaboration with Frontiers^1^. This gave us the opportunity^2^ to reach a broad audience with more than 50,000 downloads. The recorded launch event featuring Leena Pankhania (Generative AI Strategy Lead, AND Digital), María Fernanda Espinosa Garcés (former President of the United Nations General Assembly, United Nations) and the author is still available^3^. In continuation, in 2024, the WEF published its report *Top 10 Emerging Technologies of 2024*^4^. The author co-drafted chapter one on *AI for Scientific Discovery* (Fink et al., 2024). To start, here, some of the author’s thoughts in developing the document with coauthors Olga Fink (Swiss Federal Institute of Technology in Lausanne), Sang Yup Lee (Korea Advanced Institute of Science and Technology) and Andrew Maynard (Arizona State University) summarized. Beyond this, I will discuss the most recent developments in the Generative AI space and their impact on the scientific machinery. Recent surveys have begun to map the intersection of generative AI capabilities and toxicological applications, outlining both sustainability opportunities and domain-specific implementation challenges (Alam et al., 2025).

## 2 AI and large language models for scientific discovery — key issues and areas of impact

Artificial Intelligence (AI) and Large Language Models (LLMs) are transforming scientific research and enhancing the pace and scope of discovery. This section provides a high-level overview of the main domains where these tools are already reshaping practice—automated literature synthesis, hypothesis generation, experimental design, data analysis, and cross-disciplinary communication. The goal here is to introduce these areas and outline their broad significance; subsequent sections (4–7) expand on each with detailed methodologies, case studies, and domain-specific examples. These emerging technologies enable unprecedented breakthroughs by retrieving information, analyzing vast datasets, automating lab procedures, and facilitating new hypotheses through AI-driven interactions. The potential for AI extends across various domains, promising major advances in areas like disease treatment or green technologies, while at the same time, they are fostering better scientific global collaboration. As AI and LLMs integrate deeper into scientific methodologies, they not only expand the boundaries of current knowledge but also redefine how research is conducted.

### 2.1 Automated literature review and Meta-analysis transforming scientific synthesis and speeding up discovery processes

Automated literature review and the meta-analysis of the results of scientific studies using AI-driven systems represent a significant shift in how scientific knowledge is synthesized and utilized. These systems can process and analyze vast amounts of published research at speeds and scales unachievable by human efforts alone. By identifying patterns, trends, and anomalies within extensive datasets, AI can consistently offer comprehensive, up-to-date summaries and insights. This capability is especially crucial in fields like medicine and environmental science, where rapid advancements can have immediate implications on policy and practice.

One major area of impact is the enhancement of research efficiency. Now, researchers can devote more time to experimental design and data interpretation rather than sifting through literature, which is accelerating the pace of scientific discovery. These AI systems also promote a more interconnected scientific community. By breaking down barriers related to the accessibility of knowledge, they enable a democratization of information, allowing for a broader base of scientists to engage with cutting-edge research regardless of geographical or institutional boundaries.

Systematic reviews are the most objective and transparent way of synthesizing information originating from evidence-based medicine, and AI is facilitating and drastically accelerating this process. For example, Insilica’s SysRev^5^ has enabled more than 16,000 systematic review projects in the last 5 years. Equally, AI-driven platforms like Iris.ai^6^, Elicit.ai^7^ or Semantic Scholar^8^, or the ORKG ASK platform developed by the TIB Leibniz Information Centre for Science and Technology, which provides an open-source infrastructure for semantically enriched scholarly search and question answering^9^, facilitate comprehensive literature reviews by mapping out relevant studies and reducing reading time. PubMed, another example, uses AI for query manipulation, author name disambiguation, and automatic indexing of articles. PubMed for example uses AI for Best Match, query manipulation, author name disambiguation, and automatic indexing of articles (Kiester and Turp, 2022).

However, the reliance on AI for literature review also introduces challenges, including the need for open access publishing, consistently high-quality data inputs, and the potential for algorithmic biases. Ensuring the accuracy, fairness, and transparency of AI-driven reviews is essential to maintain scientific integrity and trust in AI-facilitated research methodologies. However, the value of comprehensive literature assessments and the increasingly systematic review of the body of existing knowledge is an enormous value proposition of AI-based literature analyses. A meta-analysis by Doneva et al. (2024) underscores just how far the field has leapt: powered by an LLM-centric pipeline the team screened and extracted data from more than five-hundred pharmacogenomics papers, lifting F1 scores a full ten points above rule-based baselines and completing the entire review in under a week.

### 2.2 Hypothesis generation — the interdisciplinary impact of LLMs on research

The use of LLMs for hypothesis generation marks a transformative advancement in scientific research methodologies. By analyzing existing datasets, these AI-driven systems can propose new scientific hypotheses, uncovering potential areas of investigation that might remain hidden using traditional analytical methods. This capability accelerates the discovery process and enhances the creativity and scope of scientific inquiry.

A significant benefit of LLMs in hypothesis generation is their ability to integrate and synthesize diverse data types from varied sources. This can lead to the growth of interdisciplinary research approaches, bridging gaps between distinct scientific domains and fostering innovative solutions to complex problems. Additionally, LLMs can identify subtle correlations and patterns that human researchers might overlook and propose novel hypotheses that can lead to breakthroughs in understanding and technology.

Examples of developments that highlight AI’s role in enhancing scientific understanding and innovation include, for instance, Placek et al. (2021) identify genes potentially linked to Amyotrophic Lateral Sclerosis (ALS), offering new research pathways. The paper uses sparse canonical correlation analysis (sCCA)—a machine learning method—to analyze genetic data in relation to cognitive outcomes in ALS patients. This is in enabling data-driven generation of a weighted polygenic risk score (wPRS). The use of AI to translate complex genomic data for clinicians to improve cancer treatment planning is advancing (O’Connor and McVeigh, 2025). Beyond this, a steadily growing body of AI-enabled studies now illustrates how machine-learning systems unravel scientific problems and open entirely new lines of enquiry. Jumper et al. (2021) AlphaFold-2 (Google DeepMind^10^) stunned the structural-biology community by predicting protein folds across whole proteomes with near-experimental accuracy, notably awarded recently the Nobel Prize in chemistry; laboratories that once spent months on crystallography can now begin functional studies immediately, accelerating everything from enzyme engineering to rare-disease research. In drug discovery, Stokes et al. (2020) used a deep neural network trained on > 100 million compounds to identify the antibiotic “halicin,” a scaffold wholly unlike existing classes, thereby rejuvenating the antibacterial pipeline at a time of mounting resistance (Boiko et al., 2023). Autonomous chemistry has taken a parallel leap: Boiko et al. combined a GPT-4-driven planner with robotic synthesis and analysis, demonstrating that a language-model agent can design, execute and interpret multi-step reactions without human intervention, effectively turning hypothesis generation and testing into a closed computational loop. On the translational frontier, Insilico Medicine’s generatively designed anti-fibrotic ISM001-055 advanced to Phase II trials in 2024—the first AI-invented small molecule to reach that milestone and a proof-of-concept for compressing pre-clinical timelines^11^. Materials science shows a similar pattern: DeepMind’s GNoME engine used graph neural networks to predict the stability of 380,000 hitherto unknown crystals, enlarging the searchable chemical space by almost an order of magnitude and pointing experimentalists to high-value targets for batteries and quantum devices (Merchant et al., 2023).

Together these cases—from protein folding and antibiotic discovery to autonomous synthesis, AI-invented therapeutics, crystal prediction and probabilistic weather modeling—illustrate the breadth with which data-driven methods are redefining what questions scientists can ask and how quickly they can answer them.

Google DeepMind’s newly announced AlphaEvolve^12^ agent pushes the idea of hypothesis generation beyond the life sciences (Gibney, 2025): by coupling Gemini large-language models to an evolutionary search loop that autonomously proposes, tests and refines code-based “hypotheses,” the system recently discovered a 48-multiplication algorithm for 4 × 4 complex-valued matrix multiplication—beating a record that had stood since 1969 and yielding double-digit speed-ups in critical AI kernels.

However, relying on AI for hypothesis generation also poses challenges related to their validation. Ensuring that these hypotheses are not only innovative but also scientifically valid and testable is crucial. There is also the need to address potential biases in the data used by LLMs, which could lead to skewed or unrepresentative hypotheses, impacting the direction and integrity of research efforts. While LLMs present exciting possibilities, their use must be carefully managed if we are to harness their full potential responsibly.

### 2.3 Enhancing experimental design — AI’s role in improving and optimizing experimental methodologies

AI’s role in enhancing experimental design is pivotal in streamlining research methodologies and refining the scientific process. By suggesting optimized methodologies, identifying potential pitfalls, and recommending improvements, AI systems can substantially increase the robustness and reliability of experimental outcomes. This capability is crucial across various scientific disciplines, where the complexity of experiments often poses significant challenges in design and execution.

The integration of AI in experimental design allows for a more systematic and data-driven approach to setting up experiments. AI algorithms can simulate multiple experimental scenarios in a virtual environment before actual implementation, saving time and resources while identifying the most promising approaches for investigation. An example are AI tools developed to optimize chemical reactions in pharmaceutical research, significantly reducing trial and error (Velasco et al., 2025). AI can suggest new and simpler experimental paths based on data from previous experiments (Boiko et al., 2023). This predictive capability ensures that experiments are not only well-designed but also more likely to yield valid and reproducible results. An example of this is utilizing AI applications in designing clinical trials^13^ to identify the most effective therapeutic doses with minimal side effects, as demonstrated in recent adaptive trial simulations integrating Bayesian optimization for dose-finding in oncology.

However, the use of AI in this context also raises important considerations. The quality of outcomes from AI-assisted designs depends heavily on the underlying algorithms and the data sets used to train them. Ensuring the transparency and interpretability^14^ of AI decisions is essential to in maintaining trust and understanding among researchers. Moreover, there must be a balance between AI suggestions and expert human oversight to ensure that experimental designs adhere to ethical standards and scientific rigor.

### 2.4 Data analysis and interpretation in complex research

AI’s profound impact on data analysis and interpretation in complex research fields such as genomics and epidemiology is reshaping the landscape of scientific discovery. By handling and interpreting vast datasets, AI systems enable researchers to identify patterns and relationships that are too subtle or complex for traditional analytical methods. The application of machine learning in genomics to predict disease susceptibility from vast genomic datasets represent examples (Wu et al., 2018; Ho et al., 2019; Long et al., 2023). This capability is crucial in areas where understanding the interplay of numerous genetic or environmental factors is essential to advancing knowledge and developing targeted interventions.

The integration of AI in these fields accelerates the research process, enhances the precision of findings, and supports more personalized approaches to treatment and prevention strategies. For example, in genomics, AI can predict gene function from DNA sequences, while in epidemiology, it can model disease spread and effectiveness of intervention strategies under various scenarios. Another example are AI-driven epidemiological models predicting the spread of infectious diseases like COVID-19 to inform public health decisions^15^.

However, the application of AI in data analysis also introduces significant challenges. Ensuring the accuracy and reliability of AI-generated insights requires robust algorithms and high-quality, well-curated datasets. There is also a critical need for transparency in AI methodologies to allow validation of the results by the scientific community. Moreover, ethical concerns regarding data privacy, consent, and potential biases in AI models must be rigorously addressed to maintain trust and integrity in research outcomes.

### 2.5 Improving cross-disciplinary communication by breaking down barriers and fostering global collaboration

The facilitation of cross-disciplinary communication by AI models is a critical development in the landscape of scientific research. AI’s ability to translate and clarify domain-specific terminologies and concepts enhances understanding between diverse scientific communities. This advancement is particularly vital in an era where complex global challenges require integrated approaches from multiple scientific disciplines.

AI-driven tools can bridge the communication gaps that often exist between different fields by providing accurate translations of specialized language and adapting information for interdisciplinary audiences. This enables researchers from varying backgrounds to collaborate more effectively, share insights, and innovate collectively, leading to breakthroughs that might not be achievable within single-discipline silos. Microsoft’s AI translator project^16^, which bridges language gaps in scientific collaborations, is a prominent example of AI in this context.

However, the implementation of AI in facilitating cross-disciplinary communication also introduces several challenges. Ensuring the accuracy of translations and interpretations of complex scientific terms is paramount; misinterpretations could lead to significant misunderstandings and potentially flawed research conclusions. Additionally, there is a need to maintain an appropriate level of human oversight in the integration of AI tools to preserve the nuanced understanding that expert knowledge brings to interdisciplinary collaborations. Balancing AI assistance with expert involvement is crucial to fostering productive dialogue and innovation across scientific boundaries.

## 3 From text-only models to embodied agents

The announcement of GPT-4 in March 2023 marked a watershed moment: the first general-purpose model to pass many professional examinations and to generate passable scientific prose with minimal prompt engineering. Rapid iteration followed. GPT-4o, released in May 2025, and GPT-5 in August of 2025 integrates real-time speech, vision and text processing with response latencies below three hundred milliseconds while retaining GPT-4-level reasoning accuracy^17^. Comparable leaps have come from the open-weight community. Meta’s Llama-3 series culminated in Llama-3.1, a 405-billion-parameter model that matches closed systems on standard benchmarks but—crucially—ships its weights under a permissive license, enabling transparent fine-tuning and error analysis^18^. In parallel, research groups have shifted from single-shot prompting toward *agentic chains* that decompose user goals into sequences of tool calls. OpenAI’s Deep Research framework^19^, introduced in February 2025, can retrieve, read, critique and synthesize hundreds of papers in under an hour while recording an audit trail of every intermediate step. The once-speculative idea of an “*AI PhD student*” suddenly feels proximate.

These technical breakthroughs rest on infrastructural and conceptual advances that deserve explicit mention. First, the cost of fine-tuning and inference has plummeted thanks to quantization, sparsity and specialized hardware, democratizing access for academic laboratories. Overall, from the release of ChatGPT 3.5 to the latest GPT-4o models, token inference costs have dropped by more than 10–20 times in many cases, depending on the specific tier and usage. Second, reinforcement learning from human feedback has been augmented with automated critiques and constitutional training, allowing models to align with scientific norms such as citation fidelity and hedging. Third, multimodal pre-training on cross-domain corpora—ranging from protein structures and electron-microscopy images to climate reanalysis grids—has collapsed disciplinary silos, enabling zero-shot transfer that would have required years of bespoke modeling work. Taken together these developments supply the raw capability that underlies every application discussed in the remainder of this review.

## 4 Automated literature synthesis at human-superhuman scale

Systematic reviews and meta-analyses represent the evidentiary bed-rock of modern scholarship, yet they are notorious for the months of painstaking labor they demand. The arrival of retrieval-augmented generation (RAG) architectures has transformed this landscape. In a typical workflow an LLM-powered agent issues structured search queries to multiple bibliographic databases, screens titles and abstracts against eligibility criteria, downloads full-text PDFs, extracts quantitative effect sizes and finally produces a narrative synthesis complete with forest plots and GRADE assessments. The human expert now occupies a supervisory role, validating inclusion decisions and verifying statistical calculations rather than performing every mechanical step.

Enormous gains in efficiency are being reported across medicine, psychology and materials science. At the same time, new pitfalls are emerging. LLMs can hallucinate citations or confuse similarly titled papers. RAG mitigates but does not eliminate these errors, so editorial policies must continue to insist on verifiable DOIs and transparent search strategies.

The standard-setting community has responded swiftly. An extension of the PRISMA guideline tentatively named *PRISMA-AI* is under consultation (Cacciamani et al., 2023); it aims to reflect the most pertinent technical details for reproducibility, focusing on outcomes, risk of bias, and applicability in AI-related systematic reviews and adds check-boxes for model version, temperature, retrieval index, chunk size and citation-verification method. I strongly endorse the adoption of such metadata requirements. They will protect readers and downstream meta-researchers from cryptic AI pipelines while preserving the efficiency benefits of automation.

PRISMA-AI’s principal strength lies in its capacity to embed reproducibility and transparency into AI-assisted systematic reviews by mandating detailed disclosure of model versions, retrieval indices, chunk sizes, and citation-verification methods (Cacciamani et al., 2023). This structured metadata facilitates verification, meta-research, and error tracing, enabling both human and machine auditors to assess the validity of review processes. Importantly, the framework extends the traditional PRISMA checklist to account for parameters unique to AI pipelines, such as temperature settings in large language models and retrieval augmentation strategies, which have been shown to influence inclusion decisions (Doneva et al., 2024).

However, limitations remain. Over-reliance on AI-assisted screening without adequate human oversight risks propagating selection bias or misclassification of studies, particularly when models are trained on incomplete or domain-biased corpora (Kiester and Turp, 2022). Furthermore, the absence of interoperability standards between review platforms complicates the adoption of uniform parameters across institutions. Adoption barriers include the need for targeted training in AI-specific review practices, integration of metadata requirements into journal editorial policies, and the availability of institutional infrastructure to support persistent storage of AI-generated outputs. Addressing these issues will be critical if PRISMA-AI is to transition from a consultation draft to a widely implemented reporting standard (Table 1).

**Table 1:** Comparative summary of PRISMA-AI and STARD-AI frameworks, highlighting key strengths, limitations, and potential adoption barriers.

Aspect	PRISMA-AI	STARD-AI
Strengths	Reproducibility & transparency; AI-specific metadata (model version, retrieval parameters); verifiable DOIs	Structured checklist for diagnostic AI; dataset provenance; aligns with STARD; regulatory relevance
Limitations	Risk of over-reliance; bias from incomplete corpora; lack of interoperability standards	Limited to diagnostic contexts; high compliance burden; variability in institutional readiness
Adoption Barriers	Training needs; editorial policy integration; infrastructure for metadata storage	Harmonization with other frameworks; training for authors/reviewers; phased enforcement

## 5 Hypothesis generation and the rise of computational serendipity

Popperian science proceeds by bold conjectures and severe tests, yet the generation of those conjectures is often romanticized as an act of individual genius. AI offers a complementary, data-driven route to creativity, surfacing connections that no single human mind could feasibly navigate. Multimodal foundation models trained on genomics, electronic health records, protein structures, chemical libraries and scholarly text can embed heterogeneous entities into a shared latent space. Cosine proximity in that space frequently corresponds to functional or causal relationships, suggesting drug-target pairs, gene-disease links or material-property correlations ripe for experimental validation. AI not only proves hypotheses; rather, it can accelerate the ideation phase. An illustrative example is the Acceleron system, by Nigam et al. (2024)^20^; this tool is specifically designed to assist researchers during the challenging ideation phase of the research life cycle. It employs an agent-based architecture with Large Language Models (LLMs) acting as “colleague” and “mentor” personas to guide researchers through formulating comprehensive research proposals. Acceleron aids in validating the novelty of research ideas by identifying gaps in existing literature and suggesting plausible methodologies, effectively streamlining the early stages of research development.

Closed-loop biomedical QA systems can now approach semi-autonomous literature analysis. In the WeiseEule framework by Aftab et al. (2024), a modular prompt-enhancement architecture allowed LLMs to retrieve, prioritize, and answer complex biomedical queries across large knowledge bases with minimal human input, achieving high retrieval precision and superior answer quality: In this “needle-in-the-haystack” test across 50 complex biomedical questions, automated keyword extraction, re-ranking strategies, and modularity in namespace and LLM (GPT-3, GPT-4, etc.) were used.

Sceptics rightly warn that latent-space proximity does not equate to causation and that biases in training data can skew suggested hypotheses toward well-studied pathways. The next frontier therefore lies in coupling generative modeling with causal inference tools and active-learning loops, thereby iteratively steering the agent toward genuinely novel parameter regions. The Controllable Generative Modeling via Causal Reasoning introduces the CAGE framework, which infers cause-effect relationships within the latent space of deep generative models (Bose et al., 2022). By defining and estimating unit-level causal effects, CAGE enables controllable generation based on counterfactual sampling, addressing the limitations of mere proximity in latent spaces. The next frontier in AI involves coupling generative models with causal inference tools and active learning loops to iteratively guide models toward unexplored parameter regions. Schölkopf et al. (2021) discuss how machine learning systems can move beyond statistical correlations to acquire causal representations of data, which are more robust and generalizable. They outline foundational principles and propose future directions, such as integrating causal structure learning with deep generative models.

## 6 Self-driving laboratories and autonomous experimentation

If automated literature synthesis accelerates the *reading* of science, autonomous laboratories promise to accelerate the *doing*^21^ (Tom et al., 2024; Canty et al., 2025). The archetype is the ChemCrow architecture (Bran et al., 2024), which grafts a large-language-model front-end onto a set of specialized chemistry tools—spectrometers, liquid-handling robots, chromatography systems—via an action-queue API. Give the agent a desired product and safety constraints; it searches reaction databases, proposes synthetic routes, orders reagents, schedules equipment time, executes experiments and analyses results, iteratively refining conditions until yield targets are met. In 2023 such systems were rudimentary demonstrations; by 2024 Nature reported a fully autonomous synthesis of twenty-nine organosilicon compounds, eight of which were previously unknown (Boiko et al., 2023). Recent perspectives propose shared self-driving laboratory (SDL) infrastructure, coupled with public compute-credit programs, as a means to democratize access and prevent concentration of autonomous experimentation capacity in a few well-resourced institutions (Canty et al., 2025).

The life-sciences analogue couples cell-handling robotics with high-content imaging and on-the-fly deep-learning analytics. In toxicology this means running concentration–response assays on human iPSC-derived organoids, capturing terabytes of microscopy data, extracting morphological embeddings and feeding those back into a Bayesian optimizer that decides the next dosing schedule (Renner et al., 2020; Elder et al., 2021). In vaccine research, AI has considerably compressed candidate ranking^22^ (Olawade et al., 2024). These achievements do not eliminate human scientists; they free us to focus on mechanistic interpretation and ethical oversight while machines handle the drudgery.

The regulatory implications are profound. Under the EU AI Act (European Union, 2024), any system that “*establishes level of exposure to mitigate health hazards*” is potentially high-risk if deployed outside pure research settings. Consequently, every self-driving lab targeted at translational endpoints must undergo conformity assessment, document data provenance and provide uncertainty estimates. ISO 42001 (ISO/IEC, 2023) offers a management-system framework to satisfy these obligations, analogous to ISO 13485 for medical-device quality. Early adoption will shield laboratories from costly retrofits when regulation tightens further.

## 7 Domain case studies

### 7.1 Drug discovery

The promise that AI would deliver de-novo therapeutic molecules is materializing. AI-assisted drug discovery and design is revolutionizing the pharmaceutical industry by accelerating the identification and development of new drugs. AI platforms like AtomNet use deep learning to predict molecule behavior, speeding up drug discovery processes (Zhavoronkov et al., 2019). Utilizing AI to model biochemical processes and molecular interactions enhances the efficiency and effectiveness of drug discovery, significantly reducing the time and cost typically required to bring a new drug to market. Insilico Medicine’s fibrosis candidate entered Phase II in 2024, the first small-molecule whose scaffold and pharmacophore were generated end-to-end by deep learning^23^. Their AI platform, Pharma.ai, rapidly identifies and designs novel drug candidates, specifically a promising treatment for idiopathic pulmonary fibrosis—a serious lung condition. By leveraging deep learning for simulating drug interactions and optimizing molecular structures, they significantly shortened the pre-clinical development phase. This predictive capability not only speeds up the discovery process but also improves the safety profile of developmental drugs by filtering out compounds with undesirable effects early in the process. DeepMind’s spinoff Isomorphic Labs aims to dose its first patient by late-2025^24^. These programs succeed because they combine generative chemistry with target-identification models trained on AlphaFold2 structures and public omics repositories. They also illustrate a subtle regulatory tension: AI can shave years off pre-clinical optimization, but agencies still require explainability dossiers that reconcile black-box embeddings with pharmacological intuition. Explaining *why* a molecule works is becoming as important as demonstrating *that* it works.

### 7.2 Predictive toxicology and environmental risk

AI models are transforming predictive toxicology and environmental risk assessment by enabling the prediction of the toxicological effects of chemicals with reduced reliance on physical testing. This shift not only expedites the assessment process but also enhances safety in product development and environmental protection. By simulating how chemicals interact with biological systems, AI can predict adverse effects, significantly reducing the risk to both human health and the environment. For example, in the US, the Environmental Protection Agency’s Toxicity Forecaster (ToxCast)^25^ or information on similar chemicals can be used to predict potential toxicity of chemicals using AI. A lifetime advocating for alternatives to animal testing has taught me that reproducibility and human relevance trump tradition (Hartung, 2024). AI read-across systems trained on physicochemical descriptors and curated in-vitro datasets now predict toxicity endpoints with a fidelity that rivals or exceeds in-vivo rodent studies (Luechtefeld et al., 2018; Kleinstreuer and Hartung, 2024). The use of AI in this field allows for a broader analysis of chemical safety by processing large datasets more efficiently than traditional methods. This includes integrating diverse data types—from molecular structure to real-world exposure data—to provide a comprehensive view of potential risks. Consequently, companies can refine product formulations to mitigate harmful effects before they reach the market, and regulatory bodies can make more informed decisions regarding chemical approvals. Building on recent analyses of generative AI’s potential for sustainable toxicology (Alam et al., 2025), these models can be applied to predict chemical hazards while reducing reliance on animal testing.

However, the deployment of AI in toxicology and risk assessment comes with critical considerations. The accuracy of AI predictions depends heavily on the quality and breadth of the training data. There is also a need for transparency in AI processes to build trust among stakeholders (Hartung et al., 2025), ensuring that decisions are based on interpretability and verifiable models which create dependable and reproducible results (Kleinstreuer and Hartung, 2024). Additionally, aligning AI-driven assessments with today’s regulatory standards and updating those standards to accommodate new AI capabilities are essential for realizing the full potential of AI in environmental safety.

### 7.3 Microphysiological systems and organoid intelligence

The integration of AI with microphysiological systems (Roth and MPS-WS Berlin, 2021) including organ-on-a-chip technology marks a significant advancement in biomedical research and pharmaceutical testing. Indeed, research on AI in organoids and organ-on-a-chip has increased by 1,500% over the last decade^26^, a clear indication of its perceived impact. AI enhances these technologies by enabling more accurate simulations of human organ responses, which are critical for effective disease modeling and drug testing. This synergy allows for the precise manipulation and monitoring of biological processes at the microscale, leading to better predictive models of drug efficacy and toxicity.

AI’s role in optimizing the design and operation of these systems includes automating the control of experimental conditions, analyzing large volumes of data to identify patterns, and predicting outcomes of pharmacological tests with high accuracy. This not only speeds up the research process but also reduces the reliance on animal testing by providing a more relevant human-based model.

However, the application of AI in this area also presents several challenges. Ensuring that AI systems can accurately replicate complex human physiology requires sophisticated algorithms trained on diverse and high-quality data. There are also ethical considerations related to data privacy and the potential for biases in the AI models, which could affect the reliability of the simulations. Additionally, the high costs associated with developing and maintaining advanced AI-integrated organ-on-a-chip systems may limit their accessibility and widespread adoption in the scientific community.

Organ-on-chip devices once produced informative but hard-to-quantify readouts. AI solves this by extracting rich morphological embeddings and temporal signatures from high-content imaging. Increasingly, AI allows the modeling of MPS and its fine-tuning by loops of virtual and real-world experiments (Smirnova et al., 2018). The integration of microphysiological systems with deep learning analytics has seen a substantial rise in scholarly publications over the past decade, reflecting the growing convergence of bioengineering and artificial intelligence (Hartung and Smirnova, 2025). The next horizon is so-called Organoid Intelligence (Smirnova et al., 2023): neural organoids interfaced with micro-electrode arrays whose electrical activity is modulated by reinforcement-learning algorithms. Whether such “brains in a dish” remain mere models or evolve into entities that raise moral questions is a discussion we can no longer postpone (Hartung et al., 2024).

### 7.4 Materials science

Discovery of functional materials has historically relied on intuition and costly trial-and-error. DeepMind’s GNoME project leveraged graph neural networks to propose more than 380,000 potentially stable crystal structures, dwarfing the experimental record (Merchant et al., 2023). Yet independent laboratories have so far validated fewer than 5 %, highlighting the gap between computational novelty and practical synthesis. AI remains indispensable here—particularly for inverse design conditioned on manufacturability constraints—but the GNoME episode reminds us that hype can outpace verification when metrics are ill-defined. Self-driving laboartories might help here, e.g., A-Lab was introduced as an autonomous laboratory for the solid-state synthesis of inorganic powders (Szymanski et al., 2023; Peplow, 2023).

### 7.5 Climate-scale forecasting

Beyond laboratory and molecular sciences, AI is increasingly applied to climate-scale forecasting, leveraging multimodal Earth system datasets for near-real-time prediction of extreme events and long-term climate trends. Recent advances in AI-driven climate reanalysis have demonstrated substantial gains in spatiotemporal resolution and computational efficiency, enabling more accurate forecasting of tropical cyclones, heatwaves, and precipitation extremes at global scale (Kurth et al., 2023). Such models, when integrated into the scAInce paradigm, exemplify how large-scale, machine-readable environmental data can directly inform mitigation and adaptation strategies, closing the loop between scientific prediction and policy action.

## 8 Toward governance, reproducibility and equitable access

Technological acceleration is a double-edged sword. Reproducibility crises already plague several disciplines; opaque AI pipelines risk magnifying the problem unless we embed transparency from the outset. I therefore advocate a STARD-AI checklist (Sounderajah et al., 2021), i.e., a guide for reporting studies that evaluate the accuracy of AI in diagnostic tests, that records dataset provenance, prompt parameters, temperature, tool calls and hardware details, paralleling the rigor expected for clinical trials. Publishers and grant agencies should mandate such metadata. Peer reviewers will need training, but the investment is minor compared with the societal cost of irreproducible science.

STARD-AI^27^ provides a structured checklist for transparent reporting of AI-centred diagnostic test accuracy studies, aligning with broader efforts to improve reproducibility and auditability in clinical AI research (Sounderajah et al., 2021). The framework’s strengths include its emphasis on dataset provenance, model versioning, and performance metrics, which enable peer reviewers, editors, and regulators to assess the robustness and generalisability of reported findings. By mirroring the established STARD framework, STARD-AI benefits from a familiar structure that can be more readily integrated into existing clinical research workflows (Sounderajah et al., 2021). While STARD-AI offers a mature and well-structured framework for reporting AI-centred diagnostic test accuracy studies, its current scope remains primarily confined to diagnostic applications, and broader adaptation will be needed to address non-diagnostic AI use cases in scientific research. Analogous extensions for interventional trials (CONSORT-AI) and trial protocols (SPIRIT-AI) (Liu et al., 2020a, 2020b) illustrate how domain-specific guidance can be phased into editorial and regulatory workflows (Cruz Rivera et al., 2020).

Nonetheless, its current scope is optimized for diagnostic contexts; non-diagnostic AI applications in scientific research—such as AI for hypothesis generation, laboratory automation, or predictive modeling in environmental science—may require adapted or complementary guidelines. Limitations also include the resource and time burden of completing the checklist, particularly for small research teams or early-stage projects, and variability in institutional readiness to implement such standards (Hartung et al., 2025). Potential adoption barriers include the need for harmonization with other domain-specific reporting frameworks, targeted training for authors and reviewers, and phased enforcement by journals and funding agencies to prevent compliance from becoming an exclusionary criterion.

Equity represents the other grand challenge. Access to foundation-model inference remains concentrated in a handful of corporations. Open-weight releases like Llama-3.1 demonstrate a viable alternative, yet academic researchers still face barriers when fine-tuning multi-billion-parameter models on local hardware. Cloud-credit programs and federated-learning consortia can help, but sustained public funding is indispensable. If frontier AI is to benefit global scholarship, we must treat compute as a public infrastructure akin to telescopes or synchrotrons.

To thrive in supervisory “lab-pilot” roles within AI-augmented research environments, scientists will require a broadened skillset that extends beyond traditional domain expertise, including:

- Causal inference and experimental design – to ensure AI-driven hypotheses and protocols are grounded in valid cause–effect reasoning.
- Algorithmic auditing and model interpretability – to evaluate AI outputs for bias, robustness, and reproducibility.
- Data governance and ethics – encompassing privacy, consent, FAIR compliance, and equity-of-access principles.
- Human–computer interaction (HCI) design – to optimize interfaces and workflows for collaborative human–AI decision-making.
- Cross-disciplinary communication – to translate AI-enabled insights across domains and stakeholder groups.
- Regulatory and policy literacy – to align AI-enabled research with applicable governance frameworks such as the EU AI Act and ISO 42001.

Integrating these competencies into scientific training will require deliberate curriculum reform^28^ (National Academies of Sciences, Engineering, and Medicine, 2024), particularly at the graduate level. Emerging pilot programmes—such as interdisciplinary data ethics courses at major research universities, doctoral tracks combining machine learning with biomedical applications, and professional certifications in AI governance—demonstrate viable models for embedding these skills alongside traditional disciplinary training. Building on such examples, graduate curricula could incorporate modular coursework on algorithmic accountability, causal reasoning, and HCI, coupled with practicum experiences in AI-enabled laboratories. Partnerships between universities, industry, and regulatory bodies can further ensure that training remains aligned with evolving technological capabilities and governance requirements. By institutionalizing this skillset, the next generation of scientists will be prepared not only to supervise AI-driven research effectively, but also to safeguard its integrity, inclusivity, and societal relevance.

## 9 From AI for science to science for AI: toward scAInce

The previous sections portrayed AI primarily as an instrument that accelerates existing scientific practice. A more profound transformation is now visible: scientific practice itself is being reorganized to suit the needs and affordances of AI. I call this emerging paradigm scAInce—a word fusion that signals a shift from *science powered by artificial intelligence* to *science optimized for artificial intelligence*.

The core premise of scAInce is that a machine-readable body of knowledge, rather than a corpus written solely for human eyes, will increasingly shape what counts as public knowledge. Large language models and graph neural networks learn from structured representations—tokens, triples, tensors—and their performance scales with the volume, completeness and consistency of those representations. A PDF that buries experimental details in prose is barely more digestible to a model than to a hurried reader; by contrast, a publication that exposes its methods, results and metadata in interoperable formats can be ingested, compared, critiqued and reused at scale. The consequence is a reorientation of scholarly norms toward openness, standardization and machine interpretability.

While Computational Science traditionally refers to the use of algorithmic models and numerical simulations to address domain-specific questions, and Data Science to the extraction of insights from large datasets through statistical and machine learning methods, scAInce differs in both orientation and operational logic. It treats the scientific process itself as an optimizable system for machine reasoning, reorganizing knowledge production to maximize machine interpretability and information efficiency. Whereas Computational Science and Data Science typically begin from human-posed questions and adapt tools to fit them, scAInce increasingly allows the capabilities and constraints of AI systems—such as data format requirements, model scalability, and search-space tractability—to shape which questions are asked, how they are framed, and how evidence is generated. This raises the prospect that research agendas may gradually shift from curiosity-driven exploration toward problems that are computationally convenient or yield rapid performance gains for AI. While such alignment can accelerate progress, it also risks narrowing the epistemic landscape, privileging machine-tractable inquiries over those that are societally important but algorithmically challenging. Recognizing and mitigating this bias—through governance safeguards, diversity-of-approach metrics, and deliberate preservation of exploratory science—will be essential to maintaining a balanced and inclusive research ecosystem. To clarify these distinctions and situate scAInce within the broader landscape of AI-enabled research, Table 2 compares its scope, epistemic drivers, inputs, methodological feedback loops, and associated risks with those of computational science and data science.

**Table 2:** Comparison of computational science, data science, and scAInce.

Dimension	Computational science	Data science	scAInce
Primary objective	Simulate and model complex phenomena using numerical algorithms	Extract patterns, trends, and predictions from datasets	Optimize the entire scientific process for machine interpretability and information efficiency
Epistemic driver	Human-curiosity-driven problem formulation	Data availability and statistical inference opportunities	Machine tractability, scalability, and active learning value estimates
Core inputs	Mathematical models, domain theory, and simulation parameters	Structured/unstructured datasets; statistical and ML algorithms	Machine-readable corpora, standardized metadata, interoperable knowledge graphs, megasets
Methodological feedback loop	Human designs model → computation produces results → human interprets	Human curates data → model extracts patterns → human interprets	AI identifies knowledge gaps → prioritizes experiments → integrates new data → re-optimizes research direction
Role of human expert	Model design, parameter tuning, interpretation	Data cleaning, feature engineering, interpretation	Supervisory oversight, ethical governance, methodological audit, cross-domain integration
Risks	Model oversimplification, numerical instability	Data bias, overfitting, spurious correlations	Agenda drift toward machine-tractable problems, erosion of exploratory science, concentration of data resources

### 9.1 Machine-readable publications as the default scholarly unit

An open, machine-readable publication begins with full-text that carries persistent identifiers for every cited entity—ORCID^29^ for authors, ROR^30^ for institutions, DOI^31^ for references, RRID^32^ for reagents—and continues with well-structured supplementary material. Figures link to raw image repositories; tables export as CSV (Comma-Separated Values); statistical analyses store provenance information on software version, seed and confidence intervals. The Singapore Statement on Research Integrity^33^ called for transparency as early as 2010, but until recently the incentive to comply was weak. Today compliance confers a tangible performance dividend when AI systems mine the literature, making richly annotated papers more visible, more citable and ultimately more fundable.

### 9.2 FAIR data and the primacy of metadata

The FAIR principles^34^—findable, accessible, interoperable and reusable—have become a mantra, yet in practice many “FAIR” datasets are discoverable only through bespoke portals or lack harmonized ontologies. scAInce demands stricter adherence. Metadata is no longer administrative afterthought but scientific infrastructure. Higashi et al. (2024) offer a vivid proof-point: their LLM-guided refactoring engine harmonized fourteen-million sample descriptors from the Gene Expression Omnibus with near-perfect precision, turning a notorious metadata quagmire into a fully query-able knowledge graph overnight. Without controlled vocabularies, units, provenance and licensing terms, even petabyte-scale repositories remain opaque to automated reasoning. The European Open Science Cloud^35^ has begun to enforce ontology-aligned deposition for funded projects, and major US agencies follow suit under the 2023 federal data-sharing mandate. Journals should do the same. At *Frontiers in AI* we now request a machine-readable data-availability statement as a condition of acceptance.

### 9.3 From small-scale experiments to quality-controlled megasets

A second hallmark of scAInce is the replacement of piecemeal, idiosyncratic studies by coordinated campaigns that create *megasets*: large, quality-controlled datasets whose marginal cost per additional observation is low relative to their societal value. The rationale echoes evidence-based medicine: individual trials are fragile, but systematic evidence accumulates power. In toxicology, integrated testing strategies that combine high-throughput screens, computational models and selected in-vivo assays already outperform single-study paradigms (Caloni et al., 2022). Similar trends are evident in structural biology, where the AlphaFold Protein Structure Database (Varadi et al., 2024) delivers complete proteomes, and in environmental monitoring, where global biodiversity observatories generate harmonized sensor streams. AI thrives on these megasets, and in turn it exposes residual coverage gaps, guiding where next to invest experimental resources.

### 9.4 AI as director of research: creating the missing nodes

Once knowledge is represented as a graph—nodes for entities, edges for relations—AI can compute the value of potential new nodes. Techniques borrowed from active learning and Bayesian experimental design estimate the expected information gain of a yet-to-be-performed study. Funders can therefore allocate budgets not by topical fashion or political pressure but by *information efficiency*: which experiment, if executed, would shrink overall uncertainty the most? There is enormous potential in such information-gain scoring, letting review-bots rank grant applications and, in effect, price experimental uncertainty in real time. Converging evidence confirms the feasibility of that vision: Google’s newly granted patent US 11354342 B2^36^ operationalizes an “information-gain” score for ranking documents in automated assistants, the Bayesian-experimental-design community now offers differentiable estimators of expected information gain that can steer live wet-lab protocols (Ao and Li, 2024), and the GFlowNets framework generalizes the same criterion to high-dimensional molecular and materials search—together showing that uncertainty-priced, information-efficient science already works from search engines to bench-top synthesis. The result is a virtuous cycle: comprehensive datasets feed better models; models reveal knowledge gaps; gap-driven funding yields new data that further enrich the Commons.

### 9.5 An emerging information economy for experimental studies

The epistemic currency of scAInce is machine-readable information. When data acquire clear, computable utility, market-like mechanisms become feasible. Tokenized data rights, prediction-market-inspired funding pools, and pay-for-results contracts could channel private capital into high-impact experiments while keeping outputs open. Skeptics fear enclosure of the knowledge commons, yet well-designed licenses—copyleft for data, permissive for downstream models—can reconcile investment incentives with openness. The Global Alliance for Genomics and Health^37^ has demonstrated that federated data trusts can protect individual privacy while enabling population-scale AI analyses. Similar structures could underwrite climate models, materials databases or even large-animal toxicology surrogates.

### 9.6 Impact on data-poor and qualitative disciplines

An important dimension of equity in *scAInce* relates to disciplines that are inherently data-poor or rely primarily on qualitative methods, such as parts of the humanities, anthropology, certain branches of history, and emerging scientific domains still in early data-collection phases. Because the *scAInce* paradigm thrives on large, standardized, and machine-readable “megasets,” these fields risk marginalization if their contributions are undervalued in algorithmically optimized research agendas. This could deepen existing disparities in visibility, funding, and scholarly influence, while narrowing the epistemic diversity needed for a balanced scientific ecosystem.

Several approaches can mitigate this imbalance. First, synthetic-data generation—when transparently documented—can augment sparse datasets, enabling AI models to explore plausible scenarios without displacing the interpretive richness of original sources. Structured frameworks for synthetic data creation, spanning both quantitative and qualitative domains, have recently been proposed, with applications including synthetic populations, expert systems, and survey replacements (Timpone and Yang, 2024). Second, cross-modal transfer learning from richer domains can allow models to draw inferences even from small or unstructured corpora, for example by aligning textual, visual, and metadata representations. Third, machine-assisted “quantitizing” approaches can help transform qualitative insights into analyzable formats without eroding their contextual nuance, facilitating the integration of humanities and social science data into large-scale AI systems (Karjus, 2023). Fourth, funding earmarks and policy incentives can explicitly support the digitization, annotation, and integration of qualitative archives into interoperable formats, ensuring they remain accessible to AI systems without losing their interpretive depth. Finally, governance frameworks should monitor and report discipline-level AI adoption and impact metrics to identify early signs of exclusion. Embedding such safeguards into *scAInce* infrastructures will help maintain the inclusivity, interdisciplinarity, and cultural breadth that are vital to the long-term health of the scientific enterprise.

### 9.7 A call to action

Researchers should treat machine-readable dissemination as integral to the scientific method, not as an optional post-hoc gesture. Journals must raise the bar on data and metadata deposition. Funders ought to allocate a fixed percentage of every grant to ensure FAIR compliance and to support community curators. Finally, the international policy community, building on UNESCO’s 2021 Recommendation on Open Science^38^, should negotiate an intergovernmental accord that guarantees open, machine-readable access to publicly funded research.

The reward is immense. scAInce promises an era in which collective understanding expands not by incremental labor but by compounding returns on shared information. If we seize the opportunity, AI systems will not merely replicate existing scholarship; they will help us design its scaffolding.

### 9.8 Risks and ethical considerations

Science for AI raises its own dilemmas. If citation counts already skew toward English-language journals, algorithmic optimization may amplify the imbalance, marginalizing scholars from the Global South whose work remains under-represented in major repositories. Data monopolies could emerge if access to megasets becomes paywalled or subject to opaque governance. Equally troubling is the potential erosion of serendipity: an AI that allocates funds purely by information gain might undervalue exploratory research lacking obvious immediate payoff. Governance frameworks must therefore include equity-of-access and diversity-of-approach metrics alongside efficiency scores.

A less quantifiable but equally significant concern is the potential erosion of the “spirit of science” if research agendas increasingly converge on machine-tractable problems. Left unchecked, algorithmic optimization for information gain or predictive accuracy could inadvertently deprioritize exploratory, high-risk inquiries that lack immediate computational payoff but are essential for paradigm shifts. As summarized in Table 2, scAInce differs from computational and data science in that its methodological feedback loops are often shaped by AI system capabilities; without corrective measures, this dynamic may narrow the epistemic landscape, privileging efficiency over curiosity. This narrowing of focus may reinforce existing disciplinary hierarchies, deepen inequalities between data-rich and data-poor fields, and reduce the diversity of epistemic approaches that sustains scientific resilience. To counter this tendency, governance frameworks should incorporate diversity-of-approach metrics alongside efficiency and reproducibility benchmarks, ensuring that funding and publication systems explicitly reward methodological pluralism, underrepresented research topics, and unconventional study designs. Such safeguards will help preserve the creative breadth and serendipitous discovery that have historically driven transformative advances in science.

## 10 Conclusion: from co-pilot to lab-pilot

**Figure 1:**
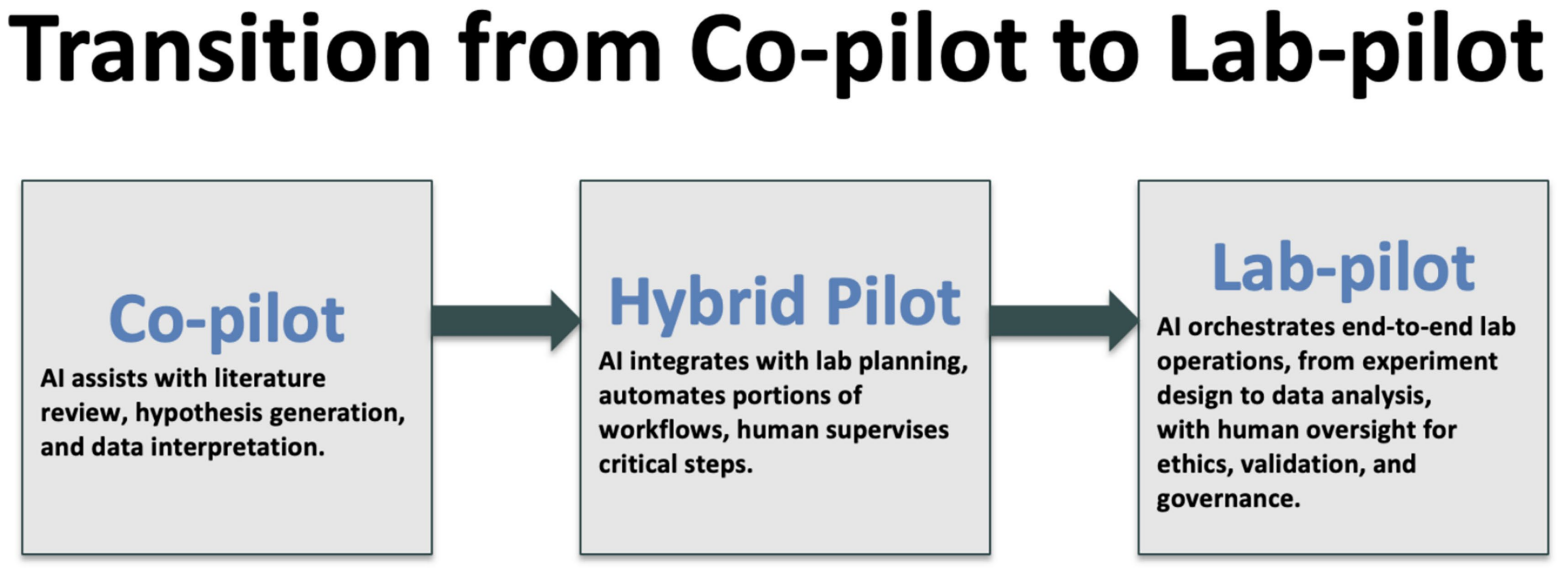
Illustration of the anticipated transition from AI as a supportive co-pilot to an orchestrator of scientific processes.

**Figure 2:**
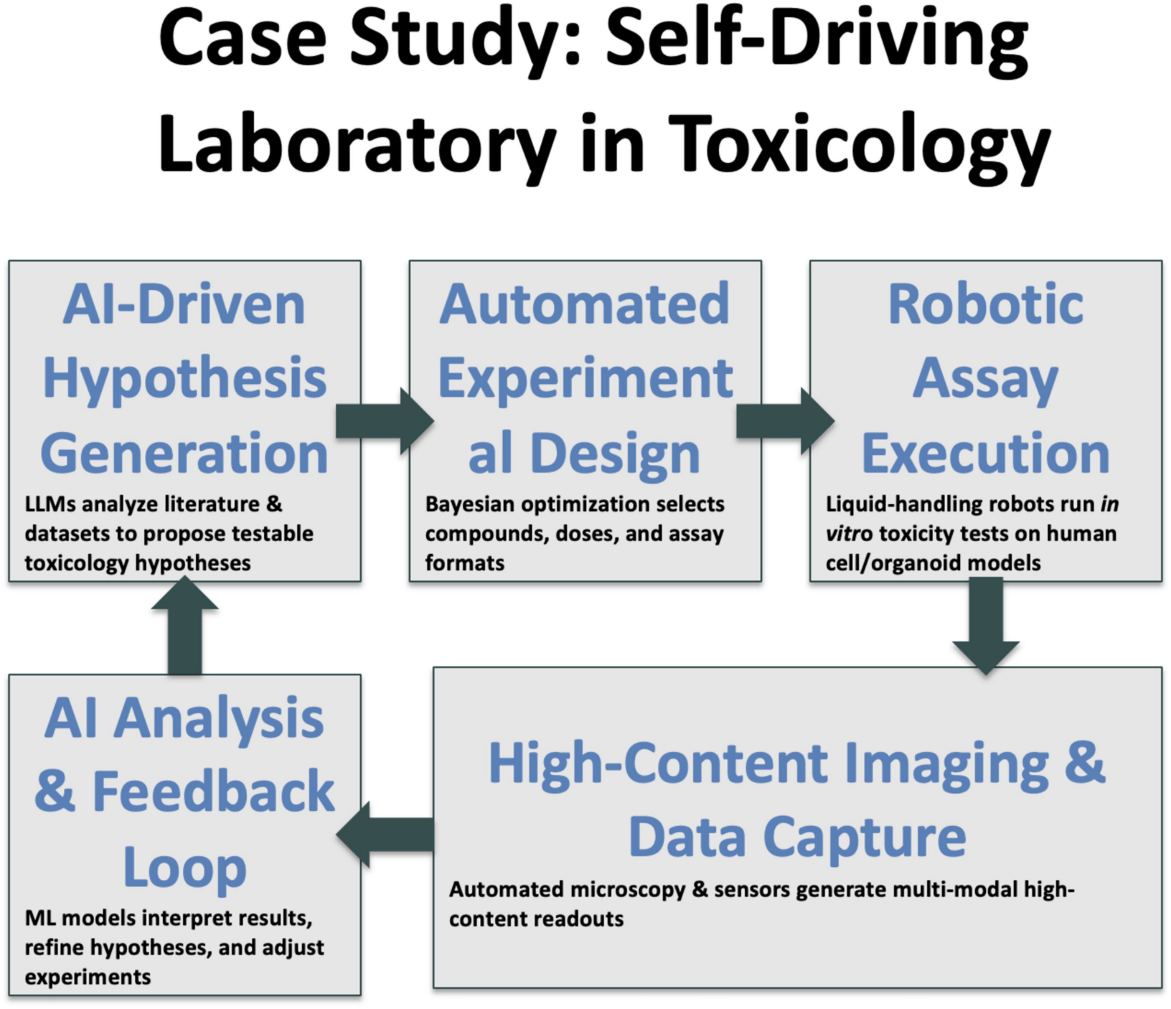
Example of the envisaged lab-pilot for toxicology.

We stand on the brink of a qualitative shift in how knowledge is generated. The distinction between *reading* science and *doing* science is blurring as agentic AI systems orchestrate both literature review and laboratory execution (Figure 1). Early evidence suggests that tasks once requiring months now compress into days without sacrificing rigor. Yet the speed advantage brings proportional responsibility. Regulators, editors and researchers must collaborate to embed transparency, safety nets and equitable access before autonomous workflows become irreversible defaults. Figure 2 gives an example of a lab-pilot to toxicology.

Seen from the vantage-point of 2025, the horizon is startlingly clear. Within a few years, expert elicitation projects such as METR^39^ (Measuring Elicitation of Technological Capabilities), using structured methods to extract knowledge from experts to make decisions or assess performance in areas where information is limited or uncertain, predict that goal-directed agents will complete month-long human research projects within a single day. I find the estimate optimistic yet believable. The critical question is not *whether* science will accelerate but *whose science* will accelerate and under what safeguards. As editors we have the privilege and the duty to shape this trajectory. Let us wield that responsibility with the same curiosity, humility and insistence on evidence that define the best of our profession.

## Footnotes

^1^https://intelligence.weforum.org/topics/a1G680000008gwUEAQ (last accessed 12 Aug 2025).

^2^https://www.frontiersin.org/news/2023/06/26/ai-in-healthcare-world-economic-forum-emerging-technologies-report (last accessed 12 Aug 2025).

^3^https://toplink.weforum.org/sessions/a0W68000008hxmUEAQ/top-10-emerging-technologies-2023? (free registration required) (last accessed 12 Aug 2025).

^4^https://www3.weforum.org/docs/WEF_Top_10_Emerging_Technologies_of_2024.pdf (last accessed 12 Aug 2025).

^5^https://www.sysrev.com (last accessed 12 Aug 2025).

^6^https://iris.ai/ (last accessed 12 Aug 2025).

^7^https://elicit.com (last accessed 12 Aug 2025).

^8^https://www.semanticscholar.org (last accessed 12 Aug 2025).

^9^https://ask.orkg.org/; source code: https://gitlab.com/TIBHannover/orkg/orkg-ask (last accessed 12 Aug 2025).

^10^Available at: https://deepmind.com/research/case-studies/alphafold (last accessed 12 Aug 2025).

^11^https://insilico.com/news/tnik-ipf-phase2a?utm_source=chatgpt.com (last accessed 12 Aug 2025).

^12^https://deepmind.google/discover/blog/alphaevolve-a-gemini-powered-coding-agent-for-designing-advanced-algorithms/ (last accessed 12 Aug 2025).

^13^https://www.clinicaltrials.gov/ (last accessed 12 Aug 2025).

^14^https://www.interpretable.ai/interpretability/what/ (last accessed 12 Aug 2025).

^15^https://covid19.healthdata.org/ (last accessed 12 Aug 2025).

^16^Available at: https://www.microsoft.com/en-us/research/blog/microsoft-research-and-worldwidescience-org-collaborate-to-remove-language-barriers/ (last accessed 12 Aug 2025).

^17^https://openai.com/index/hello-gpt-4o/ (last accessed 12 Aug 2025).

^18^https://ai.meta.com/blog/meta-llama-3-1/ (last accessed 12 Aug 2025).

^19^https://openai.com/index/introducing-deep-research/ (last accessed 12 Aug 2025).

^20^https://ai-2-ase.github.io/papers/7%5CCameraReady.pdf (last accessed 12 Aug 2025).

^21^https://www.futurehouse.org (last accessed 12 Aug 2025).

^22^https://www.mckinsey.com/industries/life-sciences/our-insights/beyond-the-pandemic-the-next-chapter-of-innovation-in-vaccines? (last accessed 12 Aug 2025).

^23^*In Silico* Medicine. (2023). “AI-Driven Drug Discovery Propels Novel Candidate into Phase 2.” Available at: https://www.insilico.com/clinical-trials and https://www.eurekalert.org/news-releases/1058384 (last accessed 12 Aug 2025).

^24^https://www.pymnts.com/artificial-intelligence-2/2025/google-deepmind-ceo-ai-designed-drugs-coming-to-clinical-trials-in-2025/?utm_source=chatgpt.com (last accessed 12 Aug 2025).

^25^https://www.epa.gov/chemical-research/toxicity-forecaster-toxcasttm-data (last accessed 12 Aug 2025).

^26^https://www.pharmiweb.jobs/article/research-on-ai-in-organoids-and-organ-on-a-chip-sees-1-500-percent-growth-in-the-last-decade- (last accessed 12 Aug 2025).

^27^FAIRsharing.org: STARD-AI; STAndards for the Reporting of Diagnostic accuracy - Artificial Intelligence, DOI: 10.25504/FAIRsharing.fffd9e, https://fairsharing.org/6247 (last accessed 12 Aug 2025).

^28^UNESCO (2021). Recommendation on Open Science. Paris: UNESCO. https://www.unesco.org/en/legal-affairs/recommendation-open-science (last accessed 12 Aug 2025).

^29^https://orcid.org (last accessed 12 Aug 2025).

^30^https://ror.org (last accessed 12 Aug 2025).

^31^https://www.doi.org (last accessed 12 Aug 2025).

^32^https://www.rrids.org (last accessed 12 Aug 2025).

^33^https://www.jsps.go.jp/file/storage/general/english/e-kousei/data/singapore_statement_EN.pdf (last accessed 12 Aug 2025).

^34^https://www.go-fair.org/fair-principles/ (last accessed 12 Aug 2025).

^35^https://research-and-innovation.ec.europa.eu/strategy/strategy-research-and-innovation/our-digital-future/open-science/european-open-science-cloud-eosc_en (last accessed 12 Aug 2025).

^36^https://patents.google.com/patent/US11354342B2/en (last accessed 12 Aug 2025).

^37^https://www.ga4gh.org (last accessed 12 Aug 2025).

^38^https://www.unesco.org/en/open-science (last accessed 12 Aug 2025).

^39^https://metr.org
